# Supplementary material for: Snord116-dependent diurnal rhythm of DNA methylation in mouse cortex
Source: Nat Commun. 2018 Apr 24;9:1616. doi: 10.1038/s41467-018-03676-0 (PMC5915486; doi:10.1038/s41467-018-03676-0)
Supplement: Supplementary file 1 — Supplementary Information(PDF 2891 kb) [file 41467_2018_3676_MOESM1_ESM.pdf]

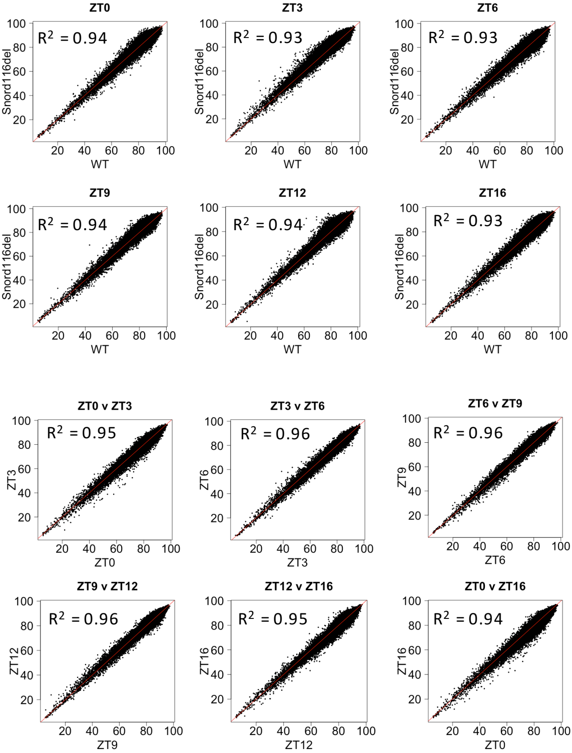

Supplementary Figure 1. Genome-wide methylation over 20 kilobase (kb) windows. *Snord116* genotype and diurnal time do not affect whole genome methylation levels when assayed in 20 kb windows.

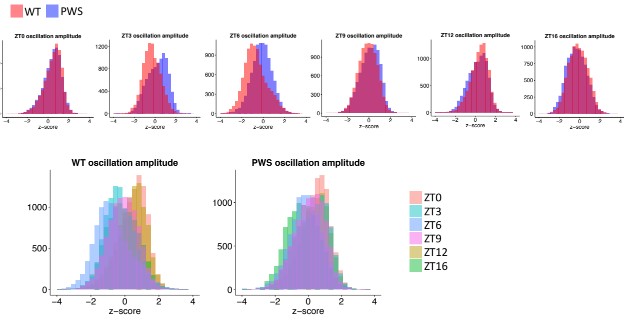

Supplementary Figure 2. Oscillation amplitude of light-phase rhythmic DMRs represented as a z-score from the overall methylation distribution for both genotypes combined. Genotype comparisons are graphed for each time point and the full time-series is graphed together for each genotype. Increased oscillation amplitude is observed at ZT3 and ZT6 in WT. No large shift to another single time point is observed in PWS. Oscillation amplitude of DNA methylation is greater across time points in WT compared to PWS.

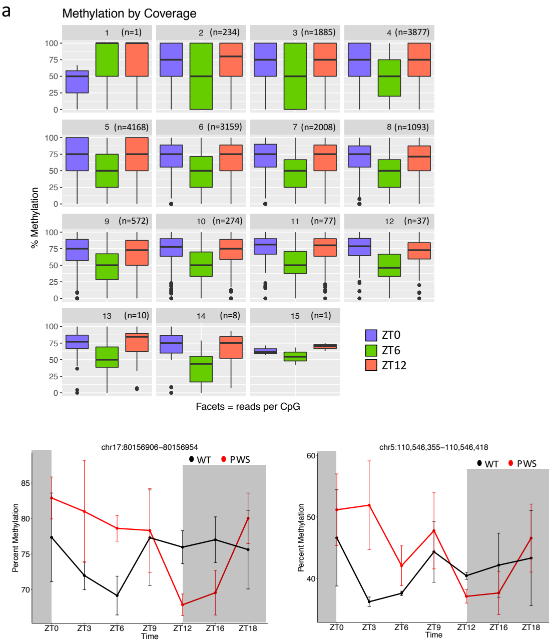

Supplementary Figure 3. Validation of rhythmic DMRs. (a) Rhythmic methylation patterns are conserved at all coverage depths observed. Each facet represents coverage, with the number of rhythmic CpGs at each coverage level indicated. (b) Two DMRs were assayed by pyrosequencing across diurnal time for each genotype, confirming the rhythmic methylation patterns observed by WGBS. N = 3 per genotype and time condition. Methylation is plotted as the average methylation of all CpGs within each DMR and error bars represent s.e.m.

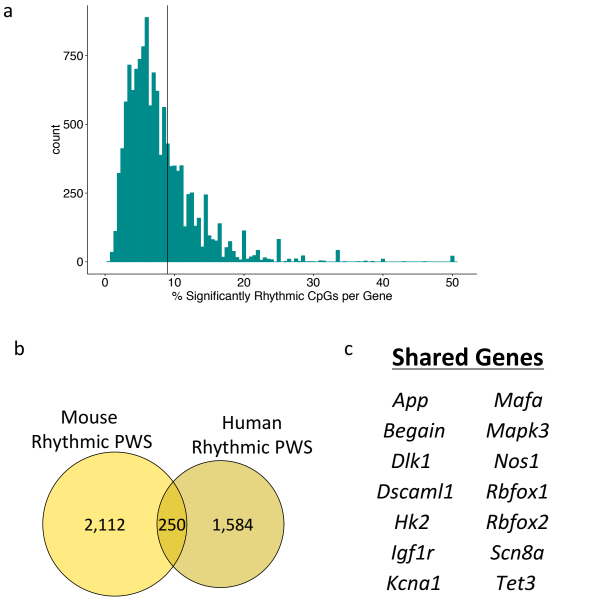

Supplementary Figure 4. Identification of rhythmic genes in human and overlap with PWS DMR genes. (a) Distribution of rhythmic CpG content per gene. Genes containing ≥9% rhythmic CpGs per gene were considered rhythmic (https://www.synapse.org/#!Synapse:syn3157275). (b) Overlap of rhythmically methylated genes disrupted in PWS between mouse and human cortex (Fisher's exact test, $p=3.3x10^{-13}$). (c) PWS rhythmic genes shared between species have functions in a variety of pathways including metabolism and sleep. See Supplementary Data 3 for full list.

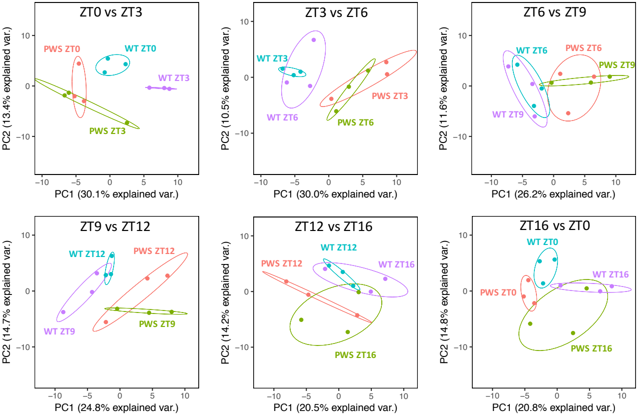

Supplementary Figure 5. Principal component analysis of WT vs PWS DMRs sampled at three-hour intervals. Ellipses represent the 95% confidence interval for each group. Non-overlapping ellipses indicate a significant difference in methylation profile ($p<0.05$). The initial early light phase change in DNA methylation profile is observed at the resolution of three-hour intervals and begins at ZT3 in WT cortex.

a

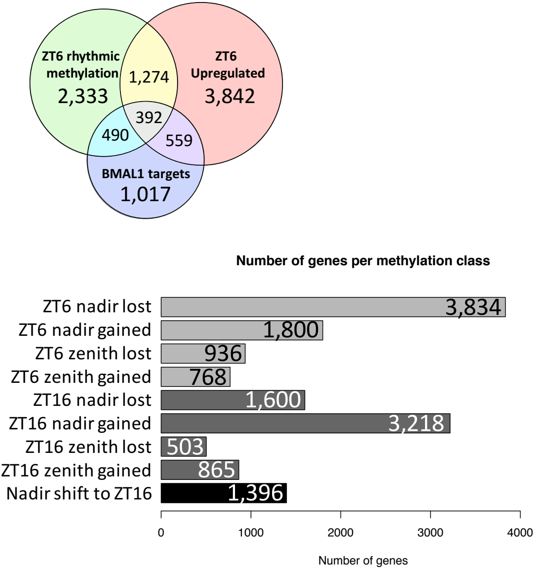

Supplementary Figure 6. Overlap of dysregulated genes with BMAL1 targets and ZT6 upregulated genes divided by diurnal methylation class. (a) Overlap between ZT6 upregulated genes, ZT6 rhythmically methylated genes, and BMAL1 targets. (b) Gene counts for each class of methylation change observed at rhythmic CpGs. Loss and gain of zeniths and nadirs are relative to the methylation state in WT. The greatest number of genes lose a nadir at ZT6 or gain a nadir at ZT16. These two groups also show the greatest overlap with genes upregulated at ZT6 in *Snord116*$^{+/-}$ cortex (Fig. 4).

a

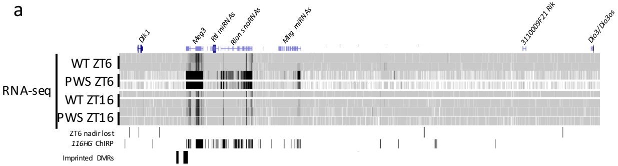

b

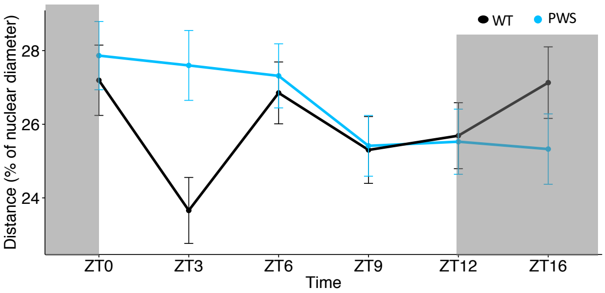

Supplementary Figure 7. Diurnal interaction between the PWS and TS loci. (a) Full *Dlk1-Dio3* locus including tracks for RNA-seq, ZT6 nadir lost DMRs, *116HG* ChIRP peaks, and known imprinted DMRs. (b) Distance between the PWS and TS loci calculated as a percent of the nuclear diameter. N = 300 nuclei from 3 cortices for each genotype and time point, error bars represent standard error of the mean (s.e.m.).

Time comparisons	n	DMRs
ZT0 vs ZT6	6 vs 6	21,129
ZT0 vs ZT12	6 vs 6	4,972
ZT0 vs ZT16	6 vs 6	20,393
ZT6 vs ZT12	6 vs 6	11,836
ZT6 vs ZT16	6 vs 6	3,540
ZT12 vs ZT16	6 vs 6	11,152

Genotype comparisons	n	DMRs
WT vs PWS	12 vs 12	275
WT vs PWS (incl ZT3 and ZT9)	18 vs 18	101
WT vs PWS, ZT0	3 vs 3	6,355
WT vs PWS, ZT3	3 vs 3	10,805
WT vs PWS, ZT6	3 vs 3	4,869
WT vs PWS, ZT9	3 vs 3	6,441
WT vs PWS, ZT12	3 vs 3	4,636
WT vs PWS, ZT16	3 vs 3	4,006

WT time comparisons	n	DMRs
WT ZT0 vs ZT3	3 vs 3	22,994
WT ZT0 vs ZT6	3 vs 3	15,747
WT ZT3 vs ZT6	3 vs 3	7,060
WT ZT3 vs ZT9	3 vs 3	6,887
WT ZT6 vs ZT9	3 vs 3	6,878
WT ZT0 vs ZT12	3 vs 3	6,593
WT ZT0 vs ZT16	3 vs 3	8,185
WT ZT6 vs ZT12	3 vs 3	9,849
WT ZT9 vs ZT12	3 vs 3	8,260
WT ZT6 vs ZT16	3 vs 3	4,254
WT ZT12 vs ZT16	3 vs 3	5,873

PWS time comparisons	n	DMRs
PWS ZT0 vs ZT3	3 vs 3	7,919
PWS ZT0 vs ZT6	3 vs 3	9,282
PWS ZT3 vs ZT6	3 vs 3	6,314
PWS ZT3 vs ZT9	3 vs 3	4,876
PWS ZT6 vs ZT9	3 vs 3	5,487
PWS ZT0 vs ZT12	3 vs 3	5,821
PWS ZT0 vs ZT16	3 vs 3	13,763
PWS ZT6 vs ZT12	3 vs 3	6,062
PWS ZT9 vs ZT12	3 vs 3	6,445
PWS ZT6 vs ZT16	3 vs 3	4,406
PWS ZT12 vs ZT16	3 vs 3	7,991

Supplementary Table 1. Summary of DMRs for each genotype and time comparison.

RNAseq ZT6 upregulated genes			fold change over expected		
			Promoter	Gene body	Enhancers
ZT6	Nadir	Lost	**0.85**	**0.83**	**3.06**
		Gained	**1.85**	**0.71**	**1.90**
	Zenith	Lost	**1.36**	**0.88**	**2.08**
		Gained	**1.35**	**0.64**	**2.38**
ZT16	Nadir	Lost	**1.64**	**0.80**	**1.92**
		Gained	**1.20**	**0.91**	**3.65**
	Zenith	Lost	**2.46**	**0.66**	**0.90**
		Gained	**1.07**	**0.90**	**1.96**

Supplementary Table 2. Genomic region enrichment analysis of PWS-specific dysregulated diurnal CpGs within ZT6 upregulated gene regions. Colored directional heat maps represent enrichment in each category with $p<4.23x10^{-3}$ (100,000 permutations) for enrichment (red) or de-enrichment (blue) shown as fold change over expected.

	Rhythmic CpGs	Non-rhythmic CpGs
WT rhythmic CpG sites	28,686 (0.39%)	7,400,159
WT null permutation 1	2,204 (0.03%)	7,475,176
WT null permutation 2	2,532 (0.03%)	7,451,527
WT null permutation 3	2,873 (0.04%)	7,432,793
PWS rhythmic CpG sites	8,417 (0.11%)	7,420,428
PWS null permutation 1	3,638 (0.05%)	7,473,742
PWS null permutation 2	2,313 (0.03%)	7,451,746
PWS null permutation 3	3,610 (0.05%)	7,432,056

Contingency Tables

	WT	Null	
Rhythmic	28,686	2,536	
Non-rhythmic	7,400,159	7,453,165	$p < 0.0001$

	PWS	Null	
Rhythmic	8,417	3,187	
Non-rhythmic	7,420,428	7,452,515	$p < 0.0001$

	WT	PWS	
Rhythmic	28,686	8,417	
Non-rhythmic	7,400,159	7,420,428	$p < 0.0001$

Supplementary Table 3. Null rhythmicity analysis by permutation of time points for WT and PWS.
