# Supplementary material for: Snord116-dependent diurnal rhythm of DNA methylation in mouse cortex
Source: Nat Commun. 2018 Apr 24;9:1616. doi: 10.1038/s41467-018-03676-0 (PMC5915486; doi:10.1038/s41467-018-03676-0)
Supplement: Supplementary file 3 — Description of Additional Supplementary Files(PDF 131 kb) [file 41467_2018_3676_MOESM3_ESM.pdf]

Description of Additional Supplementary Files

File Name: Supplementary Data 1
Description: Summary of whole genome bisulfite sequencing data

File Name: Supplementary Data 2
Description: List of WT ZT6 rhythmic DMRs.

File Name: Supplementary Data 3
Description: Gene overlaps between mouse and human PWS and rhythmic genes.

File Name: Supplementary Data 4
Description: Coordinates of rhythmically methylated CpGs for each genotype and those that are gained, lost, and maintained in *Snord116*$^{+/-}$ (PWS).

File Name: Supplementary Data 5
Description: Gene lists for CpGs with disrupted rhythmic methylation in *Snord116*$^{+/-}$ (PWS).

File Name: Supplementary Data 6
Description: Significantly enriched Homer motifs for DMRs with disrupted rhythmic methylation in *Snord116*$^{+/-}$ (PWS).

File Name: Supplementary Data 7
Description: Genes with significantly dysregulated expression in *Snord116*$^{+/-}$ (PWS).

File Name: Supplementary Data 8
Description: Pathway analysis of genes upregulated at ZT6 with disrupted rhythmic methylation in *Snord116*$^{+/-}$ (PWS). Significant KEGG and dbGaP terms including genes assigned to each term.

File Name: Supplementary Data 9
Description: Imprinted locus enrichment for CpGs with disrupted rhythmic methylation in *Snord116*$^{+/-}$ (PWS).

File Name: Supplementary Data 10
Description: All known transcription factor binding motifs found within ZT6 nadir lost DMRs within the TS locus and the number of times they are found.

File Name: Supplementary Data 11
Description: Mixed model ANOVA post-hoc test p-values, with Tukey correction, for DNA FISH measurements of the PWS and TS loci and the distance between loci from ZT0 to ZT16.
